# Validation of a multiplex flow immunoassay for detection of IgG antibodies against SARS-CoV-2 in dried blood spots

**DOI:** 10.1371/journal.pone.0252621

**Published:** 2021-05-28

**Authors:** Coleman T. Turgeon, Karen A. Sanders, Piero Rinaldo, Dane Granger, Heather Hilgart, Dietrich Matern, Elitza S. Theel

**Affiliations:** 1 Divisions of Laboratory Genetics and Genomics and Department of Laboratory Medicine and Pathology, Mayo Clinic, Rochester, Minnesota, United States of America; 2 Clinical Microbiology, Department of Laboratory Medicine and Pathology, Mayo Clinic, Rochester, Minnesota, United States of America; Waseda University: Waseda Daigaku, JAPAN

## Abstract

**Background:**

Dried blood spots (DBS) are an established specimen type for clinical testing given their low cost, ease of collection and storage, and convenient shipping capabilities through the postal system. These attributes are complementary to the expansion of SARS-CoV-2 serologic testing, which may be used to inform community seroprevalence rates.

**Methods:**

The Luminex xMAP SARS-CoV-2 Multi-Antigen assay utilizes magnetic beads labeled with three viral antigens (nucleocapsid [NC], receptor binding domain [RBD], spike S1 subunit) to detect anti-viral IgG-class antibodies, and has Food and Drug Administration (FDA) Emergency Use Authorization (EUA) for use in serum and plasma. This assay was modified for use with DBS and validated against paired sera tested by one of two reference assays: the Roche Diagnostics Elecsys anti-SARS-CoV-2 ECLIA or the Euroimmun anti-SARS-CoV-2 IgG ELISA.

**Results:**

159 paired DBS and serum specimens analyzed using the modified Luminex xMAP assay on DBS and the reference methods on serum showed an overall concordance of 96.9% (154/159). Use of multivariate pattern recognition software (CLIR) for post-analytical interpretation of the Luminex xMAP DBS assay results, instead of manufacturer provided interpretive thresholds, increased overall qualitative result concordance to 99.4% (158/159) between the modified Luminex xMAP DBS and reference results.

**Conclusions:**

Use of DBS for detection of antibodies against SARS-CoV-2 provides comparable results to those obtained using serum. DBS concordance was improved with multivariate pattern recognition software (CLIR). We demonstrate that DBS are a reliable specimen type for SARS-CoV-2 antibody detection using the modified Luminex xMAP assay.

## Introduction

Detection of antibodies against severe acute respiratory syndrome coronavirus 2 (SARS-CoV-2) continues to play a limited role for diagnosis of coronavirus disease 2019 (COVID-19), however, the interest in monitoring community or institutional seroprevalence rates remains. Currently, most serologic assays with Food and Drug Administration (FDA) Emergency Use Authorization (EUA) require venipuncture collected serum or plasma for testing. This produces a number of challenges for large scale seroprevalence studies, including the risk of potential exposures, the need for phlebotomists, venipuncture supplies, and on-site sample preparation and storage equipment. Use of dried blood spot (DBS) samples obviates these challenges, as this specimen type can be self-collected by most patients via a finger stick and can be sent directly to a testing laboratory by regular mail, minimizing healthcare worker (HCW)/patient exposure risk.

We previously validated DBS samples as an alternative to serum, for detection of IgG-class antibodies against the SARS-CoV-2 spike glycoprotein using the Euroimmun anti-SARS-CoV-2 IgG ELISA (Euroimmun, Lübeck, Germany), which has FDA EUA for use on serum or plasma [1]. The modified Euroimmun ELISA performed on DBS extracts was the backbone method used for a large scale HCW SARS-CoV-2 seroprevalence study at Mayo Clinic, where nearly 30,000 volunteer participants were screened over a 6-week period [2]. Notably however, due to a high level of false positive results generated by the Euroimmun ELISA on DBS (~ 15%), a two-tier testing approach was implemented for the Mayo Clinic employee seroprevalence study: any DBS reactive result by the Euroimmun ELISA on DBS required confirmation using a venipuncture collected serum sample tested by the Roche Diagnostics Elecsys Anti-SARS-CoV-2 Total Antibody electrochemiluminescence immunoassay (ECLIA; Roche Diagnostics, Indianapolis, IN). The Roche ECLIA assay, also with FDA EUA, detects total antibodies against the SARS-CoV-2 nucleocapsid protein (NC).

In an effort to obviate the need for confirmatory testing of patients positive by the Euroimmun ELISA on DBS samples, we validated an alternative method for this specimen type–the Luminex xMAP SARS-CoV-2 Multi-Antigen IgG assay. This is a multiplex, magnetic microsphere-based flow cytometry immunoassay with FDA EUA for use in serum and plasma, which separately measures IgG against three SARS-CoV-2 antigens–the NC, the receptor binding domain (RBD) and the spike glycoprotein S1 subunit (S1). Using paired serum and DBS samples from unique patients, we show excellent correlation of results between the modified Luminex xMAP assay performed on DBS specimens and the comparator assays with FDA EUA performed on serum (*i*.*e*., the Roche ECLIA and Euroimmun ELISA).

## Materials and methods

### Samples

Paired serum and DBS samples collected from 159 unique individuals with a median age of 44 years (range 21 to 70 years) and of whom 119 (74.8%) were female, were used for this study. Among these 159 paired samples, 139 specimens were selected and de-identified from participants of the Mayo Clinic employee seroprevalence study [2]. Paired DBS and serum samples were collected a median of 1 day apart (range: 1 to 22 days). Serum from these individuals was tested by the Roche Diagnostics Elecsys Anti-SARS-CoV-2 Total Antibody ECLIA (Roche ECLIA), which resulted as positive and negative in 42 and 97 individuals, respectively.

An additional 20 paired DBS and serum residual specimens were obtained concurrently from patients 19 to 71 days (median 24 days) following testing for SARS-CoV-2 by real-time reverse transcription polymerase chain reaction (RT-PCR) [3]. Among these 20 patients, 11 were SARS-CoV-2 RT-PCR negative and nine were positive. Serum from these 20 patients was tested by the Euroimmun anti-SARS-CoV-2 IgG ELISA (Euroimmun ELISA) and matched the SARS-CoV-2 RT-PCR results, with the exception of one sample which was RT-PCR positive/Euroimmun ELISA indeterminate. This sample was considered ‘positive’ by the Euroimmun ELISA for the purposes of this study. This study was approved by Mayo Clinic’s Institutional Review Board and included written informed consent (#20–002775).

### Materials

The Luminex xMAP SARS-CoV-2 Multi-Antigen IgG Assay kit (part number: 30–00124), which includes Multi-Antigen IgG Assay Microsphere Mix, wash buffer, multi-antigen IgG detection reagent, 96-well round bottom plate, Thermowell® 96-well plate Mylar seal, and sheath fluid (part number: 40–50000), were obtained from Luminex Corporation (Austin, TX). Artificial, SARS-CoV-2 seronegative serum was purchased from Irving Scientific (Santa Anna, CA), and 96-well polypropylene plates (Greiner C650201) were purchased from Chrom Tech (Apple Valley, MN).

#### SARS-CoV-2 antibody DBS controls

SARS-CoV-2 antibody negative and positive DBS controls were prepared by combining equal amounts of washed red blood cells from a SARS-CoV-2 seronegative donor with either artificial serum or serum positive for SARS-CoV-2 IgG antibodies by the Euroimmun anti-SARS-CoV-2 IgG ELISA. The controls were then mixed by inversion, spotted on Whatman 903 filter paper, and allowed to dry for at least 2 hours.

### Methods

With the exception of specimen type, the Luminex xMAP SARS-CoV-2 Multi-Antigen assay was used to evaluate DBS extracts per manufacturer instructions for testing of serum samples. Briefly, in a polypropylene 96-well plate, a single 3 mm punch per DBS card was extracted in 100 μL of Luminex kit-supplied wash buffer for 2 hours, rotating at 200 rpm at ambient conditions. A 50 μL aliquot of DBS extract was added to a second, opaque 96-well plate and combined with 50 μL microsphere mix, containing dyed magnetic microspheres individually coated with either SARS-CoV-2 NC, RBD or S1 antigens, and internal capture quality control (QC) microspheres for IgG, IgM and IgA. Following plate rotation at 800 rpm for 1 hour at ambient conditions, microspheres were immobilized with a magnetic plate holder and the supernatant removed by aspiration. The immobilized microspheres were washed twice with 150 μL of kit supplied wash buffer, then detection reagent (50 μL) containing fluorescently labeled anti-human IgG was added, and the plate rotated at 800 rpm for 1 hour at ambient conditions. Subsequently, microspheres were magnetically immobilized, and the supernatant removed. The immobilized microspheres were again washed twice, reconstituted to a final volume of 100 μL with wash buffer and analyzed on the Luminex 200 System, a flow-cytometry-based analyzer, which provides results for a 96-well plate within 1.5 hours.

The Roche Diagnostics Elecsys Anti-SARS-CoV-2 Total Antibody ECLIA and the Euroimmun anti-SARS-CoV-2 IgG ELISA were used to test serum samples, without modification of manufacturer instructions for use. Positive results by the Roche ECLIA require a signal-to-cut-off (S/Co) value of ≥1.0. For discordant sample analysis, the Ortho-Clinical Diagnostics anti-SARS-CoV-2 IgG chemiluminescent immunoassay (CIA; Raritan, NJ) against the S1 glycoprotein, was used on serum samples according to manufacturer instruction for use.

#### Post-analytic result interpretation

Analysis of results from the Luminex xMAP SARS-CoV-2 Multi-Antigen IgG assay for serum or plasma include assessment of total microsphere bead count and median fluorescence intensities (MFI) for each of the three SARS-CoV-2 antigens (NC, RBC, and S1), control antibody microspheres (IgG, IgA, and IgM), and the background control. The kit includes rules to interpret each set of seven results as either positive, negative or “no call” (assay failure) (xMAP® SARS-CoV-2 Multi-Antigen IgG Assay Package Insert; https://www.fda.gov/media/140256/download; last accessed 4/16/2021). A positive result requires the IgG MFI to be above the lot-specific threshold (*ie*, 700 MFI) for the NC antigen and at least one of the other two antigens (*i*.*e*., RBD and/or S1). Additionally, all antigen bead counts must be above a pre-defined threshold and all controls must meet their individual acceptance criteria. The interpretation is performed by xMAP MULTI IgG CoV-2 Assay Software once the kit-supplied cutoffs have been imported. These same criteria for serum/plasma and software were used to interpret results from the modified Luminex xMAP assay performed on DBS extracts.

Additionally, an in-house developed bioinformatics platform for multivariate pattern recognition, Collaborative Laboratory Integrated Reports (CLIR, version 2.22, https://clir.mayo.edu/), was applied to results from the modified Luminex xMAP assay performed on DBS, and results compared to those acquired using the manufacturers’ result interpretation criteria and the reference serologic assays. The process to create a single condition tool has been described previously [4]. Briefly, it consists of sequential selection of a) configuration parameters (scoring and correction factor strategies); b) high and low markers (chosen on the basis of a degree of overlap between reference and condition-specific disease ranges of less than 50%); c) marker exceptions (forced zero score when the primary marker is not abnormal), and d) customized interpretation guidelines. The threshold for an informative score is set halfway between the lowest score of a known case and zero. If one or more cases had a score of zero, common occurrence with false positive data sets, the threshold is then set at a value of 1. Tools for false positive conditions are automatically made identical to the true positive counterparts, with the only difference of condition-specific numerical threshold of likelihood of disease.

#### Statistics

Statistical analysis, including for positive and negative agreement, overall concordance and kappa coefficients were done using GraphPad Prism QuickCalcs (https://www.graphpad.com/quickcalcs/).

## Results

### Imprecision studies

Intra-assay imprecision was assessed by analyzing 10 SARS-CoV-2 antibody negative and 10 SARS-CoV-2 antibody positive DBS controls within a single run. All replicates for the negative controls were qualitatively concordant, with mean NC, RBD and S1 MFI values of 4, 5 and 3, and variation coefficients (CVs) of 30.7%, 27.2%, and 15.7%, respectively. For the SARS-CoV-2 antibody positive DBS control, all replicates were qualitatively concordant, with mean NC, RBD, and S1 MFIs of 14,008, 14,011 and 3,652, and CVs of 1.5%, 1.9%, and 5.8%, respectively. Inter-assay imprecision was assessed by analyzing SARS-CoV-2 antibody negative and positive DBS controls across 10 days. Samples were qualitatively concordant across all runs, with negative mean NC, RBD, and S1 MFIs of 21, 20 and 13 and CVs of 45.7%, 38.8%, and 10.9%, respectively. For the SARS-CoV-2 antibody positive DBS control, all replicates were qualitatively concordant with mean NC, RBD and S1 MFI values of 14,814, 12,841, and 2,196, and CVs of 9.9%, 4.9%, and 13.8%, respectively.

### Method comparison

159 paired serum and DBS specimens were tested by the Roche ECLIA (N = 139) or Euroimmun ELISA (N = 20), and the modified Luminex xMAP assay, respectively, with the serum results considered the reference standard. Qualitative result concordance between the two specimen types and the respective methods was achieved for 96.9% (154/159) of the paired samples, with a kappa coefficient of 0.928 indicating ‘almost perfect agreement’ (Table 1). The discordant paired serum and DBS specimens were collected 1 to 8 days apart. The three Luminex xMAP DBS discordant positive samples had MFI values above 700 for the NC (704, 767, 3311 MFI) and S1 (796, 5951, 6289.5 MFI) antigens only; the RBD MFI in all three samples was less than 700 (MFI range: 168–324). Among the five discordant samples, two sera had sufficient volume for analysis using the Ortho-Clinical anti-SARS-CoV-2 IgG CIA, and results from both sera tested by the CIA were concordant with the Roche ECLIA (Table 1).

**Table 1:** Result comparison between 159 paired DBS and serum samples tested by the Luminex xMAP^a^ and reference serologic assays for anti-SARS-CoV-2 antibodies.

		Roche ECLIA or Euroimmun ELISA^b^ (Serum)		PPA (95% CI)	NPA (95% CI)	OC (95% CI)	Kappa (95% CI)
		Positive	Negative	96.1% (86–99.7%)	97.2% (91.8–99.4%)	96.9% (92.7–98.9%)	0.928 (0.866–0.99)
Luminex xMAP (DBS)^a^	Positive	49	3^d^				
	Negative	2^c^	105				

Abbreviations: DBS, dried blood spot; PPA, positive percent agreement; NPA, negative percent agreement; OC, overall concordance.

^a^Luminex xMAP results on DBS were interpreted using manufacturer recommended MFI criteria for serum/plasma.

^b^139 sera were tested by the Roche ECLIA and 20 sera were tested by the Euroimmun ELISA.

^c^One sample had sufficient serum for discordant sample analysis using the Ortho-Clinical Diagnostic anti-SARS-CoV-2 IgG CIA and resulted as ‘positive’.

^d^One sample had sufficient serum for discordant analysis using the Ortho-Clinical Diagnostics anti-SARS-CoV-2 IgG CIA and resulted as ‘negative’.

**Fig 1:**
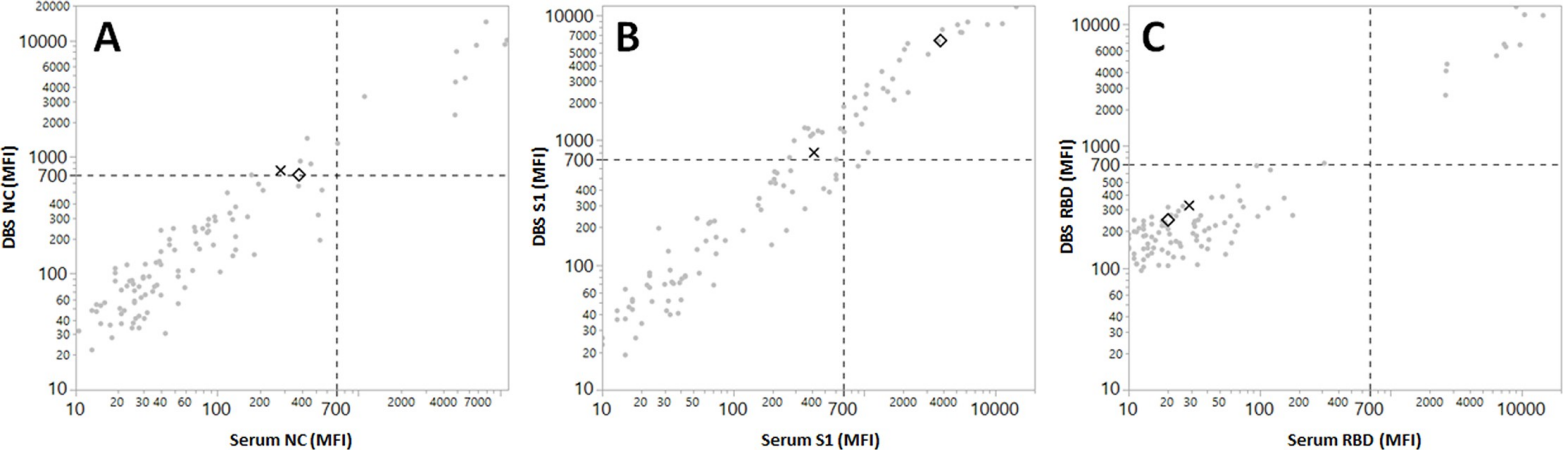
Result comparison of 107 paired DBS and serum samples analyzed for IgG against the SARS-CoV-2 nucleocapsid (NC, panel A), spike glycoprotein S1 subunit (S1, panel B) and receptor binding domain (RBD) antigens (panel C) using the Luminex xMAP assay. The samples represented by the X and the diamond indicate two cases with discordant result interpretations.

Among the 159 paired samples, 107 had sufficient serum volume for analysis using the Luminex xMAP assay per manufacturer instructions. Comparison of results from the paired DBS and serum samples, both tested by the Luminex xMAP assay, yielded 97.9% (105/107) concordance between the two specimen types (Table 2). For both of the Luminex xMAP DBS positive/serum negative discordant samples, SARS-CoV-2 antibody testing on serum by the Roche ECLIA also resulted as negative. As shown in Fig 1, the Luminex xMAP DBS positive result for one DBS was due to MFI positivity near the threshold for the NC and the S1 antigens (767 and 796 MFI). The other DBS had a near-threshold result for the NC antigen (704 MFI) while the result for the S1 antigen was significantly elevated (6290 MFI). Both discordant DBS, however, had clearly below-threshold MFI results for the RBD antigen (324 and 247 MFI).

**Table 2:** Anti-SARS-CoV-2 antibody result comparison between 107 paired DBS and serum samples tested by the Luminex xMAP assay and interpreted based on manufacturer recommended MFI threshold values.

		Luminex xMAP (Serum)		PPA (95% CI)	NPA (95% CI)	OC (95% CI)	Kappa
		Positive	Negative	100% (67.9–100%)	97.9% (92.3–99.9%)	98.1% (93–99.9%)	0.899 (0.0.761–1.0)
Luminex xMAP (DBS)	Positive	10	2^a^				
	Negative	0	95				

Abbreviations: DBS, dried blood spot; PPA, positive percent agreement; NPA, negative percent agreement; OC, overall concordance

^a^Both samples were negative by the Roche ECLIA.

Results from the 107 sera tested by the Luminex xMAP assay were also compared to the Roche ECLIA and showed 98% (105/107) overall concordance (Table 3). Among the 96 samples negative by both the Luminex xMAP and Roche ECLIA, all had NC (MFI mean: 84, range: 6–534) and RBD (MFI mean: 34, range: 6–312) antigen values below the manufacturer’s threshold (MFI 700). However, only 69 of these 96 samples had MFI levels for the S1 antigen below 700 (MFI mean: 134, range: 7–699) while the results for the remaining 27 (28%) samples were elevated (MFI mean: 3,436, range: 703–14,507). Yet, among the nine Roche ECLIA and Luminex xMAP concordantly positive samples, only one sample had an S1 antigen MFI above 700 (MFI: 900 vs. MFI mean: 357, range: 119–659, n = 8); the remaining eight samples resulted as Luminex xMAP positive due to elevated IgG against only the NC (MFI mean: 6,423, range: 711–11,197) and RBD (MFI mean: 7,861, range: 2,654–14,612) antigen beads.

**Table 3:** Result comparison between 107 serum samples tested by the Luminex xMAP and Roche anti-NC ECLIA anti-SARS-CoV-2 serologic assays.

		Roche anti-NC ECLIA (Serum)		PPA (95% CI)	NPA (95% CI)	OC (95% CI)	Kappa (95% CI)
		Positive	Negative	90% (57.4–99.9%)	99% (93.8–99.9%)	98.1% (93–99.9%)	0.89 (0.74–1.0)
Luminex xMAP (serum)	Positive	9	1				
	Negative	1	96				

### Use of CLIR software for post-analytic Luminex xMAP analysis of DBS results

For the purpose of building single condition tools with CLIR, cases were sorted in four target conditions, using the Roche ECLIA or the Euroimmun ELISA (as available per sample) as the reference standard, as follows: a) Luminex (Lu)-IgG TP, true positive cases; b) Lu-IgG FP, false positive cases; c) Lu-IgG FN, false negative cases; and d) Lu-IgG TN, true negative cases. For each of the four target conditions, post-analytical tools were generated that create a condition specific score, which is calculated from the degree of penetration for a case value into the condition range for each informative marker. Summing the scores for each informative marker yields a case score, where a threshold between 0 and the lowest case score in the training set determines if the profile is informative for a condition. When CLIR’s post-analytical interpretive tools are used in place of manufacturer provided thresholds for the modified Luminex xMAP DBS assay, 99.4% (158/159) of samples showed qualitative result concordance (Table 4). The one discordant sample resulted as positive by the Roche ECLIA with a S/Co value of 1.96, which is near the positive threshold for this assay (S/Co ≥1.0) and had an elevated Luminex xMAP DBS NC antigen MFI at 1368.5. Additional serum was not available for testing by an alternative anti-SARS-COV-2 spike glycoprotein assay for further discordant analysis.

**Table 4:** Anti-SARS-CoV-2 antibody result comparison between 159 DBS samples tested by the Luminex xMAP assay and interpreted using CLIR, and matched serum samples tested by reference serology assays.

		Roche ECLIA or Euroimmun ELISA^a^ (Serum)		PPA (95% CI)	NPA (95% CI)	OC (95% CI)	Kappa (95% CI)
		Positive	Negative	98% (88.7–100%)	100% (95.9–100%)	99.4% (96.2–100%)	0.985 (0.957–1.0)
Luminex xMAP and CLIR (DBS)	Positive	50	0				
	Negative	1	108				

Abbreviations: CLIR, Collaborative Laboratory Integrated Reports; DBS, dried blood spot; PPA, positive percent agreement; NPA, negative percent agreement; OC, overall concordance.

^a^139 sera were tested by the Roche ECLIA and 20 sera were tested by the Euroimmun ELISA.

### Distribution of NC, RBD and S1 IgG MFI values in DBS tested by the Luminex xMAP assay

**Fig 2:**
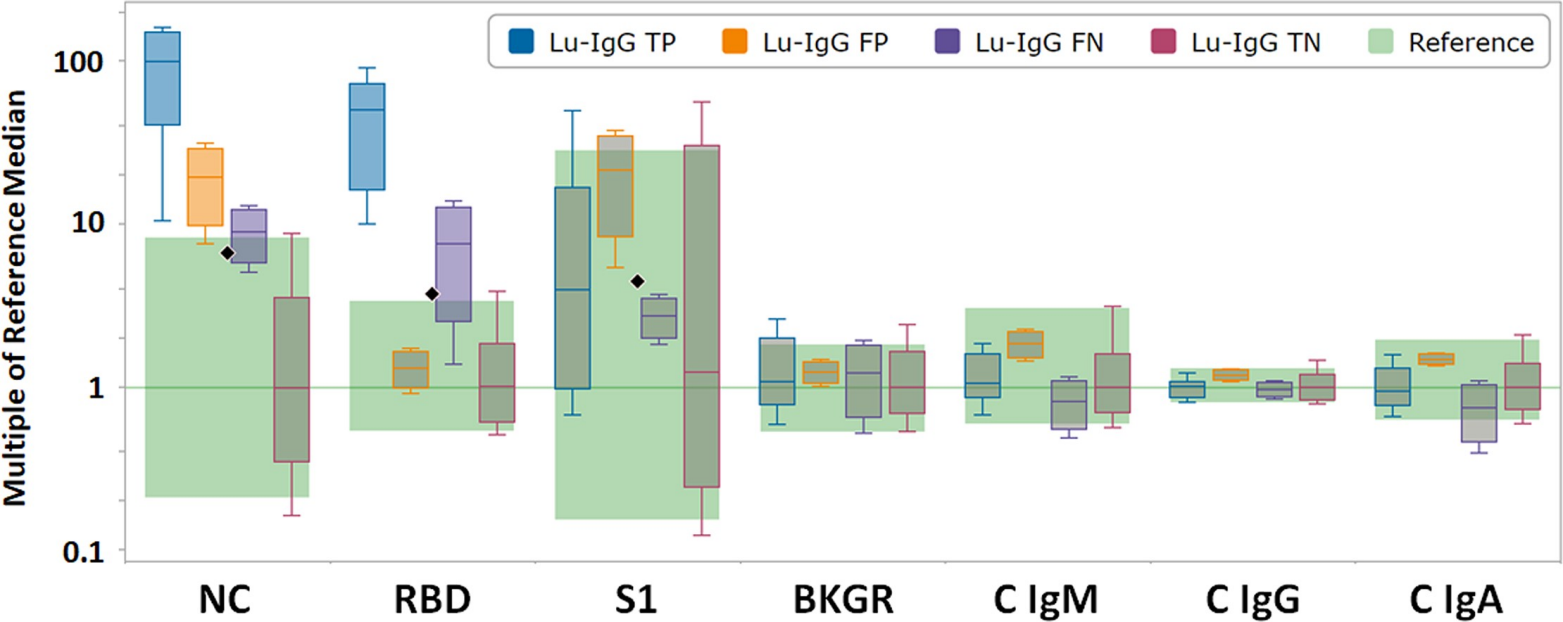
CLIR plot by multiple conditions of 159 DBS samples with serum SARS-CoV-2 antibody results. Abbreviations: NC, nucleocapsid protein; RBD, receptor binding domain; S1, spike glycoprotein S1 subunit; BGKR, background; C, control. Luminex (Lu)-IgG TP, ranges for true positive cases (N = 49; blue); Lu-IgG FP, false positive cases (N = 3; orange); Lu-IgG FN, false negative cases (N = 2; purple); and Lu-IgG TN, true negative cases (N = 105; red). Upper whisker end: 99^th^ percentile; top of box: 90^th^ percentile; line in box: median; bottom of box: 10^th^ percentile; lower whisker end: 1^st^ percentile. Black diamonds indicate the manufacturer provided cutoff (700 MFI) for IgG against SARS-CoV-2 NC, RBD and S1 antigens, respectively.

MFI reference ranges for the anti-SARS-CoV-2 NC, RBD and S1 IgG antibodies were assessed by analysis of DBS extracts from 108 SARS-CoV-2 seronegative individuals, which classified as such by using reference SARS-CoV-2 serologic assays performed on paired serum samples. Receiver operating characteristic (ROC) analysis yielded areas under the curve (AUC) of 0.99673, 0.99392, and 0.36247 for the NC, RBD and S1 antigen microspheres, respectively. Box plots of the antigen-specific MFIs are shown in Fig 2. Data were grouped according to the outcomes generated using the manufacturer’s recommended algorithm and static cutoff thresholds.

## Discussion

DBS samples are a convenient, non-invasive and self-collectable specimen type, which has been routinely applied for newborn screening since the 1960s [5] and detection of various infectious and non-infectious diseases [6–10]. Use of DBS extracts for detection of antibodies to SARS-CoV-2 is an attractive application of this sample type, as it obviates the infrastructure and supplies required for venipuncture collected blood samples and can be stored and shipped at ambient conditions through routine mail. Collectively, this makes DBS samples well suited for large-scale seroprevalence studies across diverse community settings. Previous studies with DBS samples have shown a high level accuracy using ELISA methods to detect antibodies against single SARS-CoV-2 antigens [11–14]. Notably however, these studies were primarily done using either in-house developed ELISAs without FDA EUA, or the Euroimmun anti-SARS-CoV-2 IgG ELISA. Prior experience in our laboratory using the Euroimmun ELISA on DBS extracts was associated with limited specificity (~86%) as compared to results in matched serum samples tested by an alternative, high-throughput SARS-CoV-2 serologic assay with FDA EUA [1,2]. We therefore selected to evaluate the Luminex xMAP multi-antigen SARS-CoV-2 serologic assay for use on DBS as it separately measures IgG reactivity against three viral antigens (NC, RBD and S1), which is consistent with national guidelines to assess a multi-target immune response to SARS-CoV-2, especially in low-prevalence settings, in an effort to improve the positive predictive value of serologic assays [15]. The Luminex xMAP assay was also attractive for use with DBS extracts, as the required 1:400 serum/plasma pre-dilution step suggest high assay sensitivity, which is essential given that DBS extracts are created from a 3 mm DBS punch, corresponding to approximately 3.2 μL of whole blood. For comparison, following serum/plasma pre-dilution, the final serum/plasma concentration in the test well is 0.25%, which is still approximately 10-fold higher than the final 0.032% concentration of DBS extracted whole blood as used for the modified Luminex xMAP assay.

Using paired DBS and serum samples, the Luminex xMAP assay performed on DBS extracts and interpreted using manufacturer recommended interpretive criteria, showed nearly 97% qualitative result concordance with results in matched sera tested by reference SARS-CoV-2 serologic assays which have received FDA EUA. Result concordance was further enhanced following application of CLIR for post-analytic assessment of the Luminex xMAP results on DBS. CLIR is a novel, web-based, custom-designed and coded post-analytic bioinformatics platform for the processing of laboratory data based on numerical results [4,16]. It was originally created to improve the interpretation of complex metabolite profiles, which commonly give rise to high false positive rates when static analyte cutoffs are employed. CLIR replaces analyte cutoff values with an integrated scoring algorithm that is based on the degree of overlap between reference ranges and condition-specific disease ranges. Applied to the NC, RBD and S1 MFI values from the DBS Luminex xMAP assay, CLIR improved overall result agreement with reference SARS-CoV-2 antibody results on serum to 99.4%, with only one sample remaining discordant. Notably, the single discordant sample showed elevated IgG MFI against the SARS-CoV-2 NC antigen by the Luminex xMAP assay on DBS, and was positive by the Roche ECLIA, which detects total antibodies against the NC antigen only. Although difficult to definitively resolve, this patient may have either been infected with SARS-CoV-2 at some point in the past with current low-level antibodies, or these results may indicate a false positive result by both methods.

Despite the 10-fold lower concentration of DBS extract versus serum used in the final test well, our data support use of the Luminex xMAP assay despite the poor performance characteristics for the S1 antigen as shown in Fig 1. Notably, limited utility of the S1 antigen in the Luminex xMAP assay was recently documented by Marien and colleagues, who suggest that removal of this analyte does not impact the clinical accuracy of this assay on serum [17]. Our comparative data between the Roche ECLIA and Luminex xMAP assays performed on serum support the limited reliability of antibody reactivity against the S1 antigen in the multi-plex assay, collectively suggesting that the variability observed at the S1 antigen is assay-related, rather than an artifact of using DBS as an alternative sample type for this method.

Several studies have recently been published evaluating the use of DBS specimens for SARS-CoV-2 serologic testing, using either commercial or laboratory developed ELISAs [11–14,18]. Compared to those, our approach requires the least amount of blood (3 mm DBS punch), allowing some latitude to the quality and amount of finger stick blood collected on the card, which minimizes the need for repeat collection. Additionally, use of the Luminex xMAP assay allows for increased specificity given it determines IgG reactivity against three SARS-CoV-2 antigens, versus the single viral protein used in most ELISAs. Finally, the extraction, processing and analysis of 92 patient samples and 4 controls/calibrators, can be completed within 6.5 hours by a single technologist. Using a staggered batching approach, from DBS punching to result reporting, this protocol allows for a throughput of 644 samples, completed by two technologists using one analyzer within an 8-hour shift. This compares favorably to the other recently published protocols for measurement of SARS-CoV-2 antibodies using DBS samples.

Limitations to our study include the lack of clinical characterization for all but 20 of the paired serum and DBS samples. Additionally, two different SARS-CoV-2 serologic assays were used as a combined reference standard, and although both have FDA EUA, the Euroimmun anti-SARS-CoV-2 IgG ELISA has been associated with lower overall clinical accuracy as compared to the Roche ECLIA [19,20]. Finally, this study did not undertake a thorough assessment of cross-reactivity for the modified Luminex xMAP SARS-CoV-2 assay on DBS samples. However, the manufacturer reports 100% specificity among 308 serum samples tested following collection in December 2019 (xMAP® SARS-CoV-2 Multi-Antigen IgG Assay Package Insert; https://www.fda.gov/media/140256/download; last accessed 4/16/2021), while independent studies performed through the National Institutes of Health document a specificity of 99.3% (599/603 sera; FDA: EUA Authorized Serology Test Performance; https://www.fda.gov/medical-devices/coronavirus-disease-2019-covid-19-emergency-use-authorizations-medical-devices/eua-authorized-serology-test-performance; last accessed 4/16/2021). Additionally, prior serologic assays validated for use on DBS specimens have shown similarly high specificity rates, further suggesting that it is unlikely that specificity of the Luminex xMAP SARS-CoV-2 performed on DBS would be significantly impacted [11,12].

In summary, we provide additional evidence that immunoglobulins can be extracted efficiently from small amounts of blood dried on filter paper. Although the assay has not yet been assessed in large populations, we show that use of the Luminex xMAP assay on DBS samples provides comparable results to paired serum samples tested by either the Luminex xMAP assay or alternative high-throughput SARS-CoV-2 serologic assays with FDA EUA. Considering the increasing COVID-19 vaccination rate, discrimination between natural infection and vaccine-induced seroconversion may be facilitated by use of a multi antigen assays, as current vaccines do not generate a humoral immune response to the NC protein. Therefore, detection of antibody reactivity to the RBD and S1 antigens in the absence of a response to the NC antigen, would suggest that that the immune response is due to vaccination rather than prior infection. Importantly however, although increasing data is showing that seropositive individuals are at lower risk of progression to moderate or severe COVID-19, there is as of yet no well-defined correlate of protective immunity against SARS-CoV-2 and results of antibody tests should be interpreted with caution [21,22]. Additionally, detection of an IgG response to multiple SARS-CoV-2 antigens allows for the application of bioinformatics tools such as CLIR, to optimize assay performance and reliability. The Luminex xMAP workflow for DBS extracts is amenable to high-throughput testing, and alongside the positive attributes of DBS (*e*.*g*., self-collection, direct submission to clinical laboratories by routine postal service, reduced sample collection costs, minimized SARS-CoV-2 HCW and patient exposure risks, etc.) presents an attractive and safe means to perform community or institutional seroprevalence studies.
